# Analysis of ceRNA networks and identification of potential drug targets for drug-resistant leukemia cell K562/ADR

**DOI:** 10.7717/peerj.11429

**Published:** 2021-05-25

**Authors:** Zhaoping Liu, Yanyan Wang, Zhenru Xu, Shunling Yuan, Yanglin Ou, Zeyu Luo, Feng Wen, Jing Liu, Ji Zhang

**Affiliations:** 1 Department of Clinical Laboratory, Shenzhen Traditional Chinese Medicine Hospital, Shenzhen, Guangdong, China; 2 Department of Clinical Laboratory, The First Affiliated Hospital, University of South China, Hengyang, Hunan, China; 3 Department of Clinical Laboratory, The Second Affiliated Hospital, Hainan Medical University, Haikou, Hainan, China; 4 Department of Hematology, The First Affiliated Hospital, University of South China, Hengyang, Hunan, China; 5 Molecular Biology Research Center & Center for Medical Genetics, School of Life Sciences, Central South University, Changsha, Hunan, China

**Keywords:** Long non-coding RNA, ceRNA network, Drug resistance, Bioinformatics, Leukemia

## Abstract

**Background:**

Drug resistance is the main obstacle in the treatment of leukemia. As a member of the competitive endogenous RNA (ceRNA) mechanism, underlying roles of lncRNA are rarely reported in drug-resistant leukemia cells.

**Methods:**

The gene expression profiles of lncRNAs and mRNAs in doxorubicin-resistant K562/ADR and sensitive K562 cells were established by RNA sequencing (RNA-seq). Expression of differentially expressed lncRNAs (DElncRNAs) and DEmRNAs was validated by qRT-PCR. The potential biological functions of DElncRNAs targets were identified by GO and KEGG pathway enrichment analyses, and the lncRNA-miRNA-mRNA ceRNA network was further constructed. K562/ADR cells were transfected with CCDC26 and LINC01515 siRNAs to detect the mRNA levels of GLRX5 and DICER1, respectively. The cell survival rate after transfection was detected by CCK-8 assay.

**Results:**

The ceRNA network was composed of 409 lncRNA-miRNA pairs and 306 miRNA-mRNA pairs based on 67 DElncRNAs, 58 DEmiRNAs and 192 DEmRNAs. Knockdown of CCDC26 and LINC01515 increased the sensitivity of K562/ADR cells to doxorubicin and significantly reduced the half-maximal inhibitory concentration (IC_50_) of doxorubicin. Furthermore, knockdown of GLRX5 and DICER1 increased the sensitivity of K562/ADR cells to doxorubicin and significantly reduced the IC_50_ of doxorubicin.

**Conclusions:**

The ceRNA regulatory networks may play important roles in drug resistance of leukemia cells. CCDC26/miR-140-5p/GLRX5 and LINC01515/miR-425-5p/DICER1 may be potential targets for drug resistance in K562/ADR cells. This study provides a promising strategy to overcome drug resistance and deepens the understanding of the ceRNA regulatory mechanism related to drug resistance in CML cells.

## Introduction

Multidrug resistance (MDR) can be caused by one kind of chemotherapeutic drug, and it can also result in resistance to other chemotherapeutic drugs with distinct structures and mechanisms of action. This phenomenon is common in various cancers after long-term chemotherapy, which makes cancers more and more difficult to treat (Assaraf et al., 2019; Wu et al., 2014). Leukemia is not only a malignant clonal disease of hematopoietic stem cells (HSCs), but also a fatal cancer, which seriously threatens human health (Wang et al., 2018b). Chronic myeloid leukemia (CML) is a myeloproliferative disease characterized by Philadelphia (Ph) chromosome and BCR-ABL oncogene (An et al., 2010; Jabbour & Kantarjian, 2020). Although TKIs such as imatinib are promising for the treatment of CML, there are still a large proportion of patients in blast crisis (BC) refractory or resistant to TKIs (de Lavallade et al., 2008). Combining doxorubicin, vincristine or some other drugs with imatinib or dasatinib is effective for treating BC-CML (Strati et al., 2014). It is worth noting that there are two main mechanisms of TKIs resistance: BCR-ABL-dependent and BCR-ABL-independent mechanisms (Rumjanek, Vidal & Maia, 2013). BCR-ABL-independent mechanisms include overexpression of ABCB1/MDR1/P-gp. P-gp can bind to lipophilic imatinib. Therefore, the role of MDR1 in imatinib resistance has attracted great attention (Gurney et al., 2007). The ATP-binding-cassette transporter (ABC transporter) and the solute carrier (SLC) are considered as the most related transporters to MDR. They affect the absorption, distribution, metabolism, excretion and toxicity of TKIs. In this study, the results of mRNA-seq showed that ABCB1 is significantly upregulated and ranked first in K562/ADR cells compared with K562 cells. Moreover, K562/ADR cell line is considered as multidrug-resistant cell line (Genovese et al., 2017; Zhang et al., 2013). Therefore, clarifying the molecular mechanism of MDR in leukemia through K562 ADR cell model is conducive to the development of new drugs and therapies and improve the therapeutic effect in leukemia.

Long non-coding RNA (lncRNA) is defined as a non-coding RNA longer than 200 nucleotides (Yi et al., 2016). It controls gene expression at various levels during physiological and developmental process, including epigenetic modification, transcription and scaffold assembly, and is a key factor in determining the cell fate. LncRNA plays pivotal roles in drug resistance of numerous cancers, but the detailed mechanism is still poorly understood (Liu et al., 2021; Lu et al., 2017; Smallegan & Rinn, 2019). LncRNA is composed of introns and other fragments, and its length can reach thousands of nucleotides, which establishes a good basis for adsorption and binding of a large number of microRNAs (miRNAs) (Ma et al., 2019b; Zhang et al., 2018a). LncRNA, like a sponge, can buffer and inhibit the ability of the target mRNA to encode proteins by competitively binding with miRNA response elements (MREs) on mRNA, which is called the competitive endogenous RNA (ceRNA) mechanism (Qi et al., 2020; Wang et al., 2019). Liu et al. (2019) analyzed the relationship between ceRNA mechanism and drug resistance in many types of cancers, and found that the imbalance of ceRNA networks may be one of the mechanisms leading to drug resistance. In addition, recent studies have shown that lncRNA UCA1 promotes imatinib resistance by acting as a ceRNA of miR-16 in CML cells (Xiao et al., 2017).

To explore the role of ceRNA mechanism underlying drug-resistant leukemia cells, we analyzed RNA sequencing (RNA-seq) data of doxorubicin-resistant K562 (K562/ADR) cells and doxorubicin-sensitive K562 cells, and screened out the differentially expressed lncRNAs (DElncRNAs) and mRNAs (DEmRNAs). We predicted the possible biological functions of DElncRNAs’ targets and potential signaling pathways by Gene Ontology (GO) and Kyoto Encyclopedia of Genes and Genomes (KEGG) analyses, and constructed a lncRNA-miRNA-mRNA co-expression network to further explore the potential ceRNA regulatory mechanism related to drug resistance in CML. What’s more, our results have indicated that CCDC26/miR-140-5p/GLRX5 and LINC01515/miR-425-5p/DICER1 axes might play potential regulatory roles in resistance to doxorubicin through ceRNA networks, which provides a basis for the discovery of new therapeutic targets for overcoming drug resistance and novel chemotherapeutic drugs, and has clinical potential for further research in this field.

## Materials & Methods

### Cell culture

Doxorubicin-sensitive K562 and doxorubicin-resistant K562 (K562/ADR) cells were kindly provided by Prof Jing Liu’s group from the Molecular Biology Research Center & Center for Medical Genetics, School of Life Sciences, Central South University. K562 and K562/ADR cells were maintained in RPMI-1640 medium (Hyclone, MA, USA) with 10% fetal bovine serum (Gibco, NY, USA). One μg/mL of doxorubicin was added to maintain drug resistance of K562/ADR cells and was withdrawn 1 day before the experiment. All cells were cultured in an incubator with 5% CO_2_ at 37 °C.

### SiRNA transfection

K562/ADR cells in the logarithmic growth phase were inoculated into 12-well plates at 2.5 × 10^5^ cells per well. Transfection was conducted with Ribo FECT™ CP reagent (Ribo, Guangzhou, China) according to the manufacturer’s instructions in RPMI-1640 culture medium without serum and antibiotic. The final concentration of siRNAs was 100 nM. Six hours after transfection, we continued to culture the cells in the medium containing serum for at least 48 h before the subsequent experiments. The transfection efficiency was determined by quantitative real-time PCR (qRT-PCR). All siRNAs were purchased from Genepharma (Suzhou, China). SiRNA sequences are listed in Table 1.

**Table 1:** Sequences of siRNAs used in this study.

Gene name	Sequence
si-CCDC26-1	5′-GGAUAUGUCAAUCUCACAATT-3′
si-CCDC26-2	5′-GCCGAAGACUGCAUUUCAATT-3′
si-LINC01515-1	5′-GTTGAATGTTGATGCTGTATT-3′
si-LINC01515-2	5′-GGGTGTCAGCTCTACCAAATT-3′
si-LINC01419-1	5′-CGUAACUUCCCUCAAAGCAACAACC-3′
si-LINC01419-2	5′-GUAACUUCCCUCAAAGCAATT-3′
si-GLRX5-1	5′-CTGGTGTTCGGGCTAAGAATA-3′
si-GLRX5-2	5′-CTGTATTATGATATTGCTGTA-3′
si-DICER1-1	5′-GCACAUCAAGGUGCUACUATT-3′
si-DICER1-2	5′-GCCAAGGAAAUCAGCUAAATT-3′

### Screening of DElncRNAs and DEmRNAs

RNA was isolated with Trizol reagent (Invitrogen, CA, USA) according to the manufacturer’s protocol. Agarose gel electrophoresis was performed to analyze the integrity of sample RNA and whether there was DNA contamination. The preparation and deep sequencing of the whole transcriptome library were performed by Novogene (Beijing, China). Having been constructed, the library was preliminarily quantified through Qubit 2.0 and diluted to 1 ng/μL. Then, the library’s insert size was detected with Agilent 2100 and the effective concentration was accurately quantified to ensure the quality of library (the effective concentration > 2 nM). After the library was qualified, RNA-seq was conducted on the Illumina Hi-Seq 4000 sequencing platform, and paired-end reads of 150 bp were created according to the protocol. Before screening, Cuffmerge software was first used to splice each sample. The transcripts were merged, and the transcripts with uncertain chain directions were removed to obtain the complete transcriptome information of this sequencing. Afterwards, the combined transcript collection was screened for lncRNA. Different types of transcripts (lncRNA and mRNA) were analyzed. The screening threshold of volcano plots was set as |log_2_ (fold change)| > 1 and *q*-value < 0.05 by default. The Fragments Per Kilobase of transcript per Million Fragments (FPKM) value of the differential transcripts under different experimental conditions was used as the expression level for hierarchical clustering.

### Quantitative real-time PCR

The total RNA was reverse transcribed with SuperScript III reverse transcriptase (Invitrogen, NY, USA). qRT-PCR was performed using LightCycler® 96 real-time fluorescent quantitative PCR instrument and SYBR Green I Master (Roche, Germany). The cycling conditions were 95 °C for 5 min, followed by 40 cycles at 95 °C for 10 s and cycles at 60 °C for 1 min. GAPDH served as an internal standardized control. The expression levels of lncRNA and mRNA were quantified by normalization to the endogenous GAPDH expression level by the 2^−∆∆CT^ method. Table 2 lists the specific primers used in qRT-PCR.

**Table 2:** Sequences of the specific primers used in qRT-PCR.

Gene name	Primer sequence	
LINC01515	Forward	5′-CGTGGTCGTGGAATGGACAAGG-3′
	Reverse	5′-GTGCTAACTGCAACTAAGGTGTGC-3′
CCDC26	Forward	5′-ATGGACCTGAATGAGCTGCAC-3′
	Reverse	5′-ACAGCCCAGGCTTGGTAGTT-3′
LINC01419	Forward	5′-AGCCAGCGAGACCACGAACC-3′
	Reverse	5′-TAAGGTGGCGTGTCTGGAGTCTG-3′
LINC00857	Forward	5′-CGATCACCATGCCAGGACAAGAC-3′
	Reverse	5′-TGAGGAGATCCAGTGGTACTCTGC-3′
DUXAP8	Forward	5′-ATGGAGTGGCAGTCTCTACAGGA-3′
	Reverse	5′-GTGAGTGACTCTGAGTGTGGAAGC-3′
LINC02506	Forward	5′-CTGAAGCCAAGGAGACCACGAAC-3′
	Reverse	5′-GACCTTCGCAGTGAGTGCTACAG-3′
LINC01029	Forward	5′-ACCTGAGCTGGACTCTTGCTT-3′
	Reverse	5′-CCACCGATGCTGATGTGGAAT-3′
Lnc-ALX1	Forward	5′-TCTTCTGTCAACCTGGTGCA-3′
	Reverse	5′-GTTGATTCAGGAACCCAGGG-3′
ZEB1-AS1	Forward	5′-GAGAGGCTAGAAGTTCCGCTTGC-3′
	Reverse	5′-TTAGCTCTGAGTCCTGCCACCTAG-3′
ABCB1	Forward	5′-AAATTGGCTTGACAAGTTGTATATGG-3′
	Reverse	5′-CACCAGCATCATGAGAGGAAGTC-3′
DMTF1	Forward	5′-GTCTGAACCGGCCTTTGTTTG-3′
	Reverse	5′-GCCCAGTCATTGCCATGCT-3′
GNG12	Forward	5′-GCAAAACAGCAAGCACCAAC-3′
	Reverse	5′-CTATCAGCAAAGGGTCACTCC-3′
MS4A4A	Forward	5′-TCTTGAAGGGAGAACCCAAAG-3′
	Reverse	5′-CCCCAAATTGTGTACCCGATA-3′
MARCKS	Forward	5′-TTTTTTCGAACTACACTTGGGCT-3′
	Reverse	5′-GGGTGGAAAAGTCGAGCACA-3′
CROT	Forward	5′-CGGATACGTTTATTCAGCTTGC-3′
	Reverse	5′-CCACCTCACTGCTTCAACTG-3′
TSPAN5	Forward	5′-CTCACGGCGGGCGTTCTTGC-3′
	Reverse	5′-CGAATGCCCCACAGCACTGCC-3′
DICER1	Forward	5′-GGTGGTCCACGAGTCACAAT-3′
	Reverse	5′-TAGCACTGCCTTCGTTTCGT-3′
GLRX5	Forward	5′-AAGGACAAGGTGGTGGTCTT-3′
	Reverse	5′-CTGCAGGAGAATGTCACAGC-3′
GAPDH	Forward	5′-TGGTATCGTGGAAGGACTC-3′
	Reverse	5′-TGGTATCGTGGAAGGACTC-3′

### GO enrichment and KEGG pathway analyses

The co-locations of lncRNA and protein-coding genes were used to predict their biological functions (Le et al., 2019; Le, Yapp & Yeh, 2019). The co-location threshold was set as 100 kb upstream and downstream of lncRNA. Pearson correlation coefficient was employed to analyze the correlation between lncRNA and mRNA, and mRNA genes with an absolute correlation coefficient value of 0.95 were selected for functional enrichment analysis to predict the biological functions of lncRNA. The DElncRNA target genes were used for GO and KEGG enrichment analyses with David and KOBAS software respectively. Furthermore, mRNAs enriched in the ceRNA network were analyzed with GO and KEGG (Table S1).

### Construction of lncRNA-miRNA-mRNA regulatory network

The relationship between DElncRNAs and DEmiRNAs was explored by the database starBase v3.0 based on CLIP-seq data research and the database miRcode based on the GENCODE database (Kong et al., 2020). TargetScan, miRcode and MiRanda were further used to decode the interaction between DEmiRNAs and DEmRNAs. Finally, according to the interaction between lncRNA, miRNA and mRNA, a ceRNA regulatory network was constructed and visualized by Cytoscape 3.6.1.

### Cell viability assay

Cell counting kit-8 assay (CCK-8) (Bimake, Houston, TX, USA) was used to monitor the cell viability with its provided protocol. Transfected cells were seeded into 96-well plates at 8 × 10^3^ cells per well. Doxorubicin was added to cells at concentrations of 0, 0.5, 1, 2, 4 and 8 μg/mL, and incubated for 48 h. Then 10 μL/well of CCK-8 reagent was added to the 96-well plates. Two hours later, the absorbance was measured by the microplate reader (ELX800; BioTek, Winooski, VT, USA) at wavelength of 450 nm. The IC_50_ value was determined by GraphPad Prism 7.0. All experiments were independently repeated three times.

### Statistical analysis

We analyzed all data with SPSS 22.0 and presented them by GraphPad Prism 7.0. All data were indicated as mean ± SD. We compared differences between two independent groups with Student’s *t*-test. Correlations between different parameters were analyzed using Spearman’s rank correlation test. Independent experiments were repeated three times. *P*-value < 0.05 was considered statistically significant.

## Results

### DElncRNAs and DEmRNAs between K562/ADR and K562 cells

**Figure 1:**
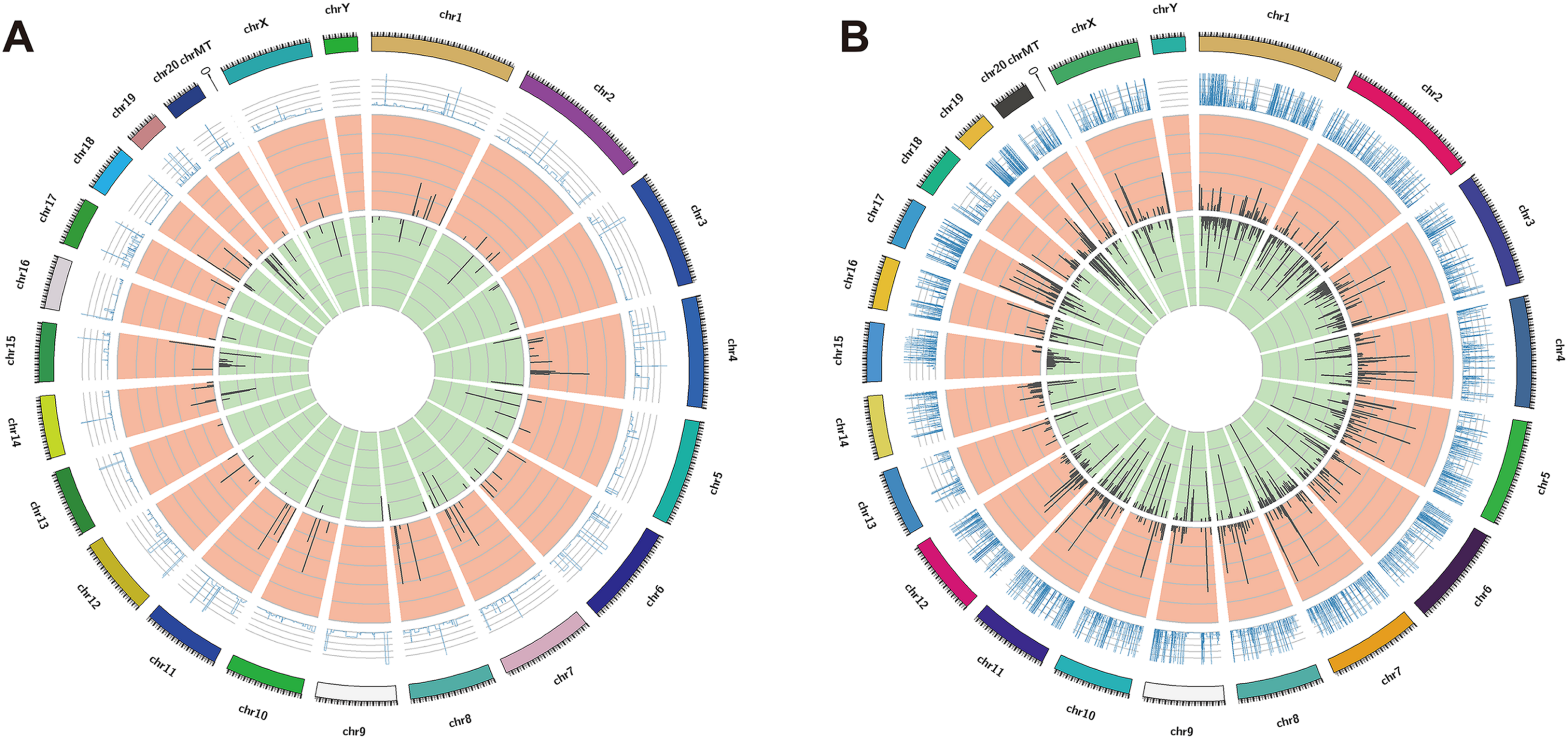
The distribution of DElncRNAs and DEmRNAs transcripts on chromosomes. All the transcripts of DElncRNAs (A) and DEmRNAs (B) were mapped to the reference genome, respectively. The global abundances on chromosomes were visualized based on the sample expression. The chromosomal distribution of differential transcripts: the outermost circle represents the chromosomes; the second circle is the sample FPKM of the paired sequences on the chromosome; the third circle represents the distribution of significantly upregulated transcripts on chromosomes; the fourth circle is the distribution of significantly downregulated transcripts on chromosomes.

**Figure 2:**
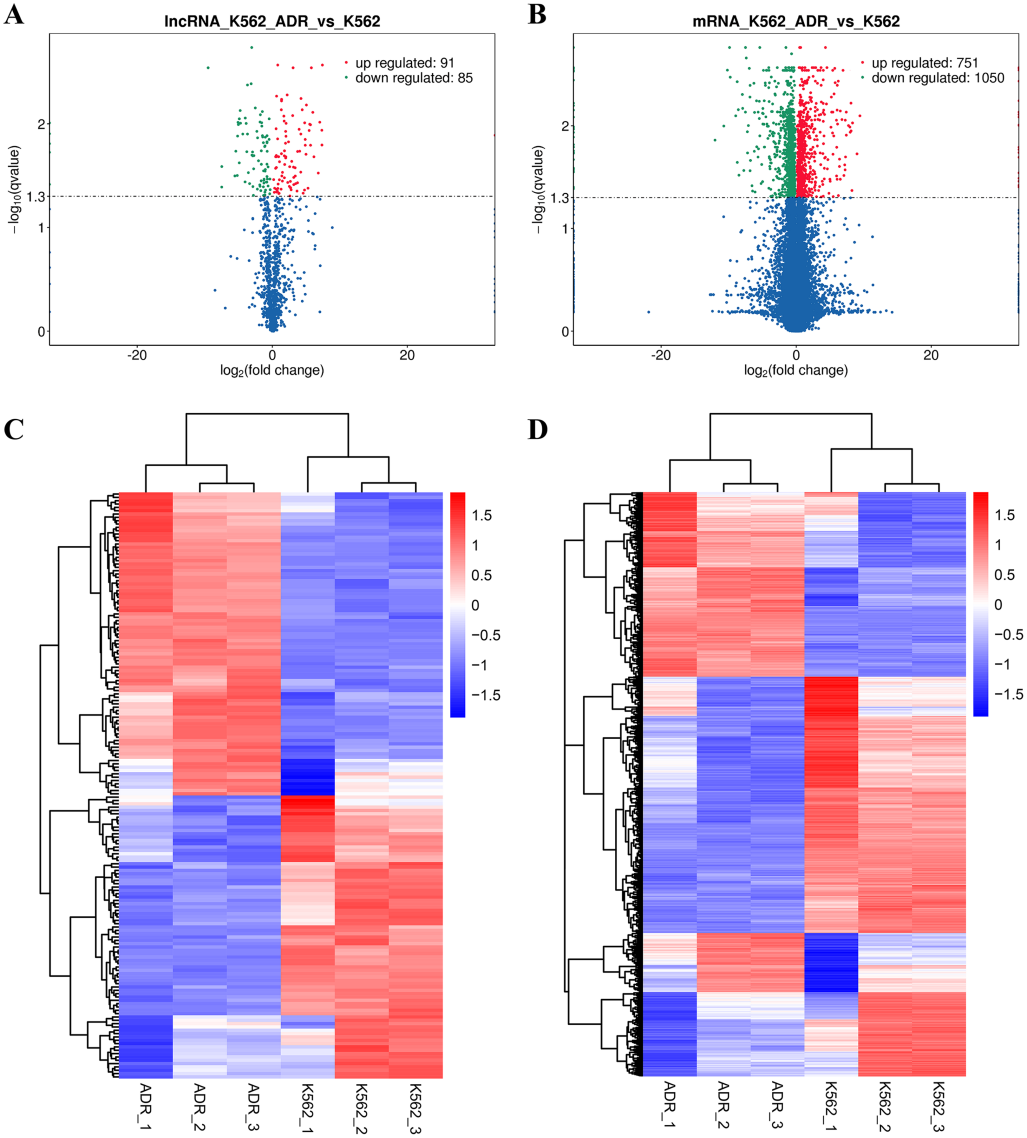
Volcano plots and heatmaps are presenting for DElncRNAs and DEmRNAs between K562 and K562/ADR cell lines. Volcano plots for DElncRNAs (A) and DEmRNAs (B) directly show the overall distribution of differential transcripts or genes, and the screening thresholds were set as |log_2_(fold change)| >1and *q*-value < 0.05 by default. The overall FPKM hierarchical-clustering-diagrams show DElncRNAs (C) and DEmRNAs (D) that were clustered by the value of log10 (FPKM+1). The red and the blue indicate the up- and downregulated genes, respectively. The color ranges from red to blue, indicating that the log10 (FPKM+1) is from large to small.

The DElncRNAs between drug-resistant K562/ADR and sensitive K562 cell lines were screened through RNA-seq conducted by Novogene. Usually, genes are distributed regularly on chromosomes, and genes close to each other may perform similar biological functions or participate in the same metabolic pathways. We visualized the global abundances of lncRNAs and mRNAs on different chromosomes based on sample expression through mapping all transcripts to the human reference genome (Figs. 1A and 1B). The differences of lncRNAs and mRNAs expression between K562/ADR and K562 cells were visualized by volcano plots (Figs. 2A and 2B) and cluster heatmaps (|log_2_ (fold change)| > 1 and *q*-value < 0.05) (Figs. 2C and 2D). Results have shown that a total of 176 dysregulated lncRNAs were identified. Among them, 91 lncRNAs were upregulated in K562/ADR cells compared with K562 cells, while the other 85 lncRNAs were downregulated. The 1801 DEmRNAs in K562/ADR cells compared with K562 cells included 751 upregulated mRNAs and 1,050 downregulated mRNAs. The top 10 upregulated and downregulated lncRNAs are listed in Table S2, moreover, the top 20 upregulated and downregulated mRNAs are shown in Table S3. These results reflect the differential expression profiles of lncRNAs and mRNAs between K562/ADR and K562 cells, implying that lncRNAs may play significant roles in the mechanism of drug resistance in CML induced by K562/ADR cells.

### Validation of lncRNA and mRNA expression by qRT-PCR

**Figure 3:**
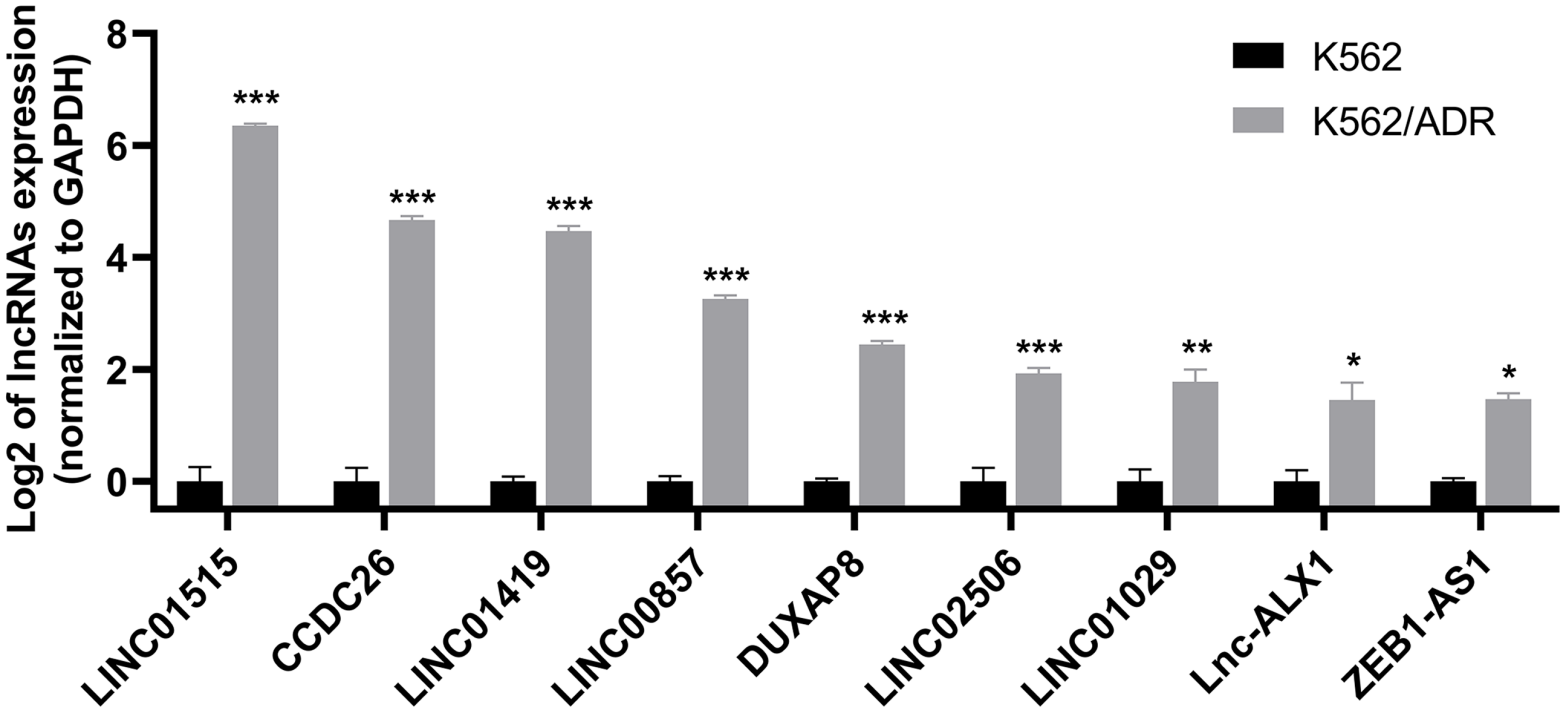
qRT-PCR was used to validate the expression of lncRNAs in the drug-resistant K562/ADR cell line. All data are represented as mean ± SD for at least three independent experiments. *P < 0.05, **P < 0.01 and ***P < 0.001 were statistically significant compared with the control group.

Nine lncRNAs and nine mRNAs were randomly chosen to validate the RNA-seq data by qRT-PCR. The results suggested that the expression of these lncRNAs (LINC01515, CCDC26, LINC01419, LINC00857, DUXAP8, LINC02506, LINC01029, lnc-ALX1 and ZEB1-AS1) and mRNAs (ABCB1, DMTF1, CROT, MS4A4A, GNG12, MARCKS, DICER1, GLRX5 and TSPAN5) were upregulated in K562/ADR cells compared with K562 cells (Fig. 3 and Fig. S1), which was consistent with the RNA-seq results, indicating that the RNA-seq data was highly reliable.

### GO and KEGG pathway enrichment analyses of target genes of DElncRNAs

**Figure 4:**
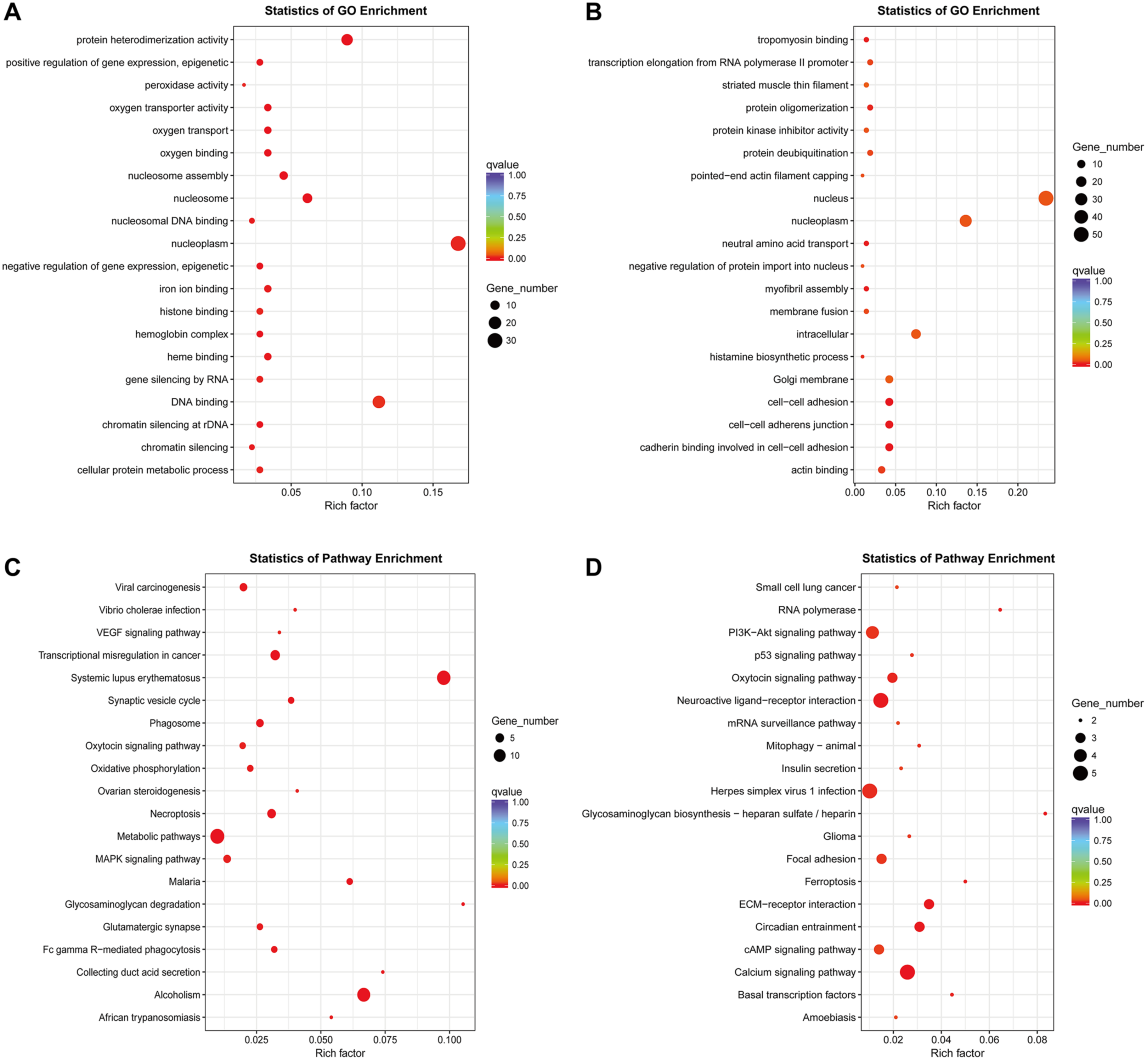
GO enrichment and KEGG pathway analyses of target genes of DElncRNAs between K562 and K562/ADR cell lines. The thresholds of co-location were set as 100 kb up- and downstream of lncRNAs. GO enrichment analysis was performed on target genes of upregulated (A) and downregulated (B) lncRNAs. KEGG pathway analysis was performed on target genes of upregulated (C) and downregulated (D) lncRNAs. The horizontal axis represents the proportion of candidate gene sets to background genes, the size of dots indicates the number of DElncRNAs targets in this pathway, and the color of dots corresponds to different *q*-value ranges.

So far, the function of lncRNA has not been fully elucidated. But previous studies have shown that part of lncRNAs interact with proteins to regulate chromatin modification, transcription and pre-mRNA splicing, and act as scaffolds for protein complex assembly (Liu et al., 2018). To further understand the mechanism of lncRNA involving in MDR-leukemia cells, the co-located targets of DElncRNAs were analyzed by GO and KEGG pathway enrichment analyses. The results of GO enrichment analysis showed that the target genes of upregulated DElncRNAs were mainly enriched in nucleoplasm, nucleosome, nucleosome assembly, regulation of gene expression, epigenetic, and cellular protein metabolic process. Target genes of downregulated DElncRNAs were mainly involved in nucleus, nucleoplasm, cell-cell adhesion, cadherin binding involved in cell-cell adhesion and cell-cell adherens junction (Figs. 4A and 4B). Additionally, KEGG pathway analysis suggested that target genes of DElncRNAs were significantly enriched in cancer-related signaling pathways, such as the MAPK, VEGF, PI3K/Akt and p53 signaling pathways (Figs. 4C and 4D).

### Construction of lncRNA-miRNA-mRNA co-expression network in drug-resistant K562/ADR cells based on bioinformatics prediction

**Figure 5:**
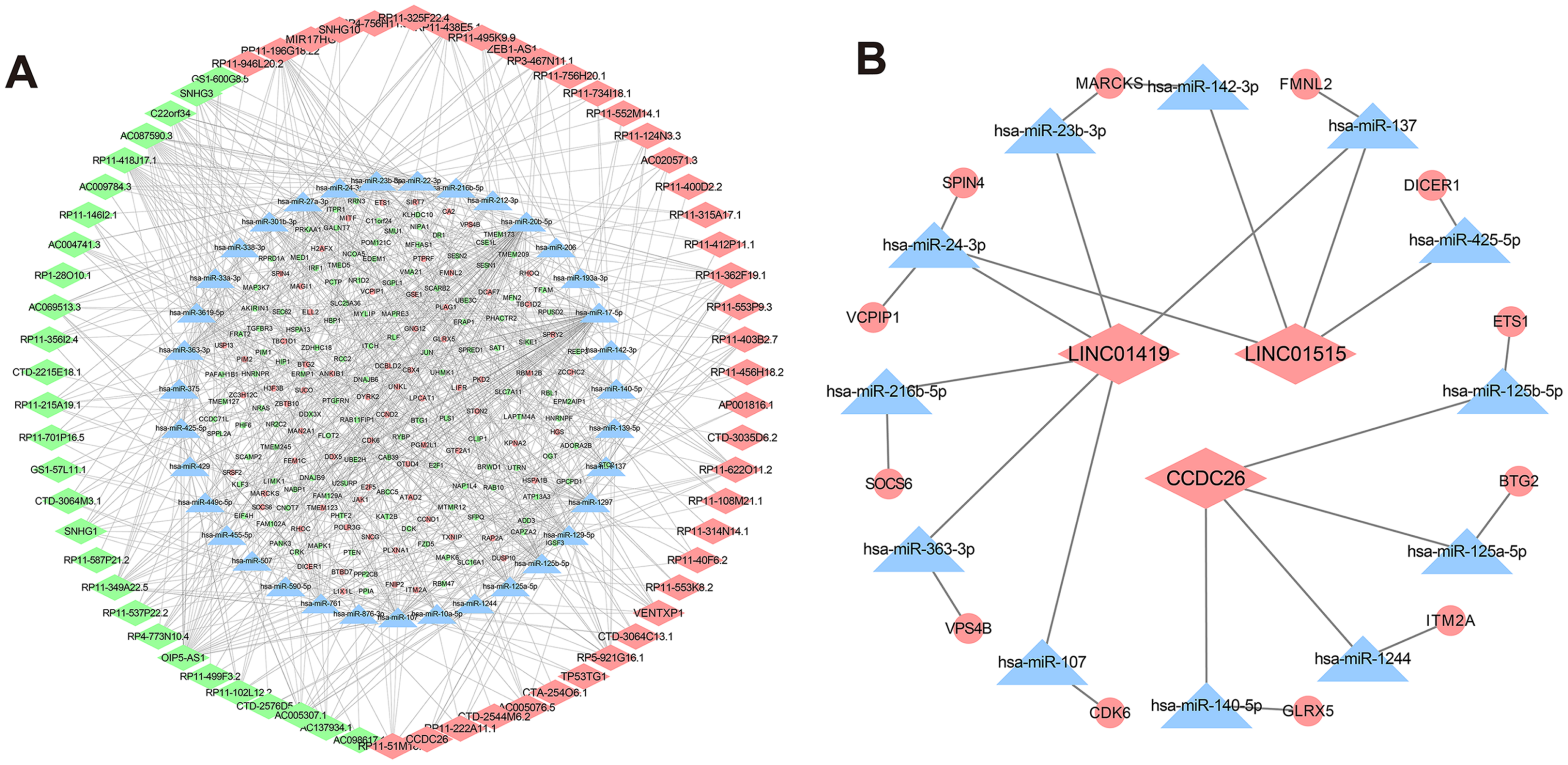
CeRNA networks in the K562/ADR cell line. Analysis of lncRNA-miRNA-mRNA ceRNA networks in the K562/ADR cell line (A). The ceRNA networks of LINC01419, CCDC26 and LINC01515 in the K562/ADR cell line (B). The diamond represents the lncRNA, triangle, the miRNA, circle represents the mRNA. All shapes in red and green represent up- and downregulation, respectively. Shapes in blue represents that upregulation or downregulation is unknown.

**Figure 6:**
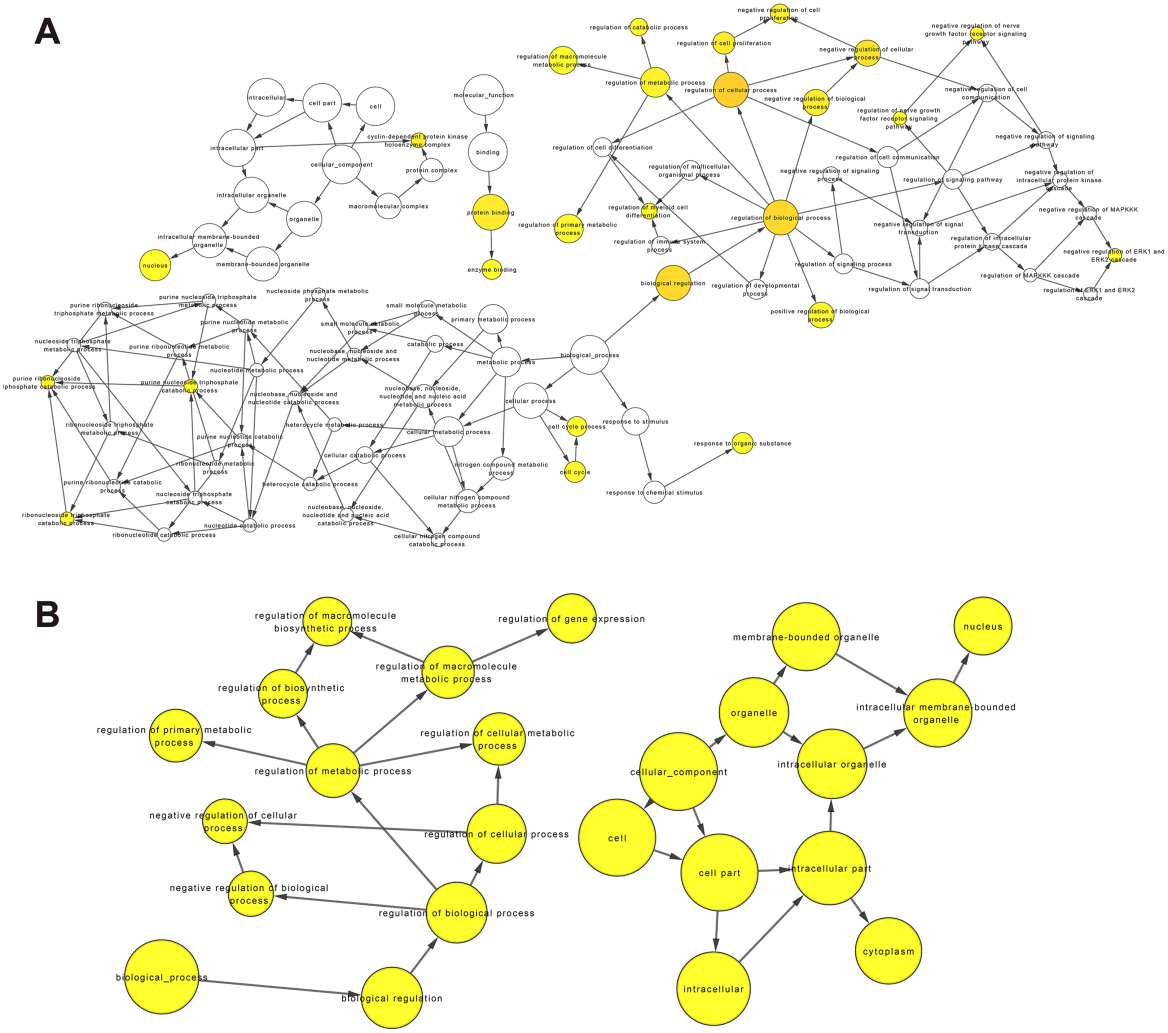
GO enrichment analysis of DEmRNAs related to the K562/ADR cell line. GO enrichment analysis was performed on upregulated (A) and downregulated (B) DEmRNAs. The shape size is proportional to the number of genes in the pathway. The color from light to dark represents the *P*-value from large to small.

Recently, complex interactions between lncRNA and miRNA have been disclosed. LncRNA can act as a miRNA sponge in the cytoplasm, thereby regulating mRNA expression (Huarte, 2015). In order to explore the mechanism of the involvement of lncRNA in drug resistance, we constructed a ceRNA interaction network composed of 409 lncRNA-miRNA pairs and 306 miRNA-mRNA pairs based on 67 DElncRNAs, 58 DEmiRNAs and 192 DEmRNAs, which was visualized with Cytoscape software (Fig. 5A). Furthermore, we focused on three DElncRNAs (CCDC26, LINC01515 and LINC01419) with the highest logFC in K562/ADR cells compared with K562 cells (Fig. 5B). To further explore the functions of DEmRNAs in drug resistance of leukemia cells, we conducted the functional enrichment analysis of them with the BiNGO plugin. As a result, GO enrichment analysis (Figs. 6A and 6B) showed that upregulated DEmRNAs were mainly enriched in cytoplasm, nucleoplasm, extracellular exosome, negative regulation of transcription from RNA polymerase II promoter and RNA binding. Downregulated DEmRNAs were mainly involved in nucleoplasm, ATP binding, poly (A) RNA binding, membrane and Golgi apparatus.

### Knockdown of CCDC26 and LINC01515 can enhance the sensitivity of drug-resistant K562/ADR cells

**Figure 7:**
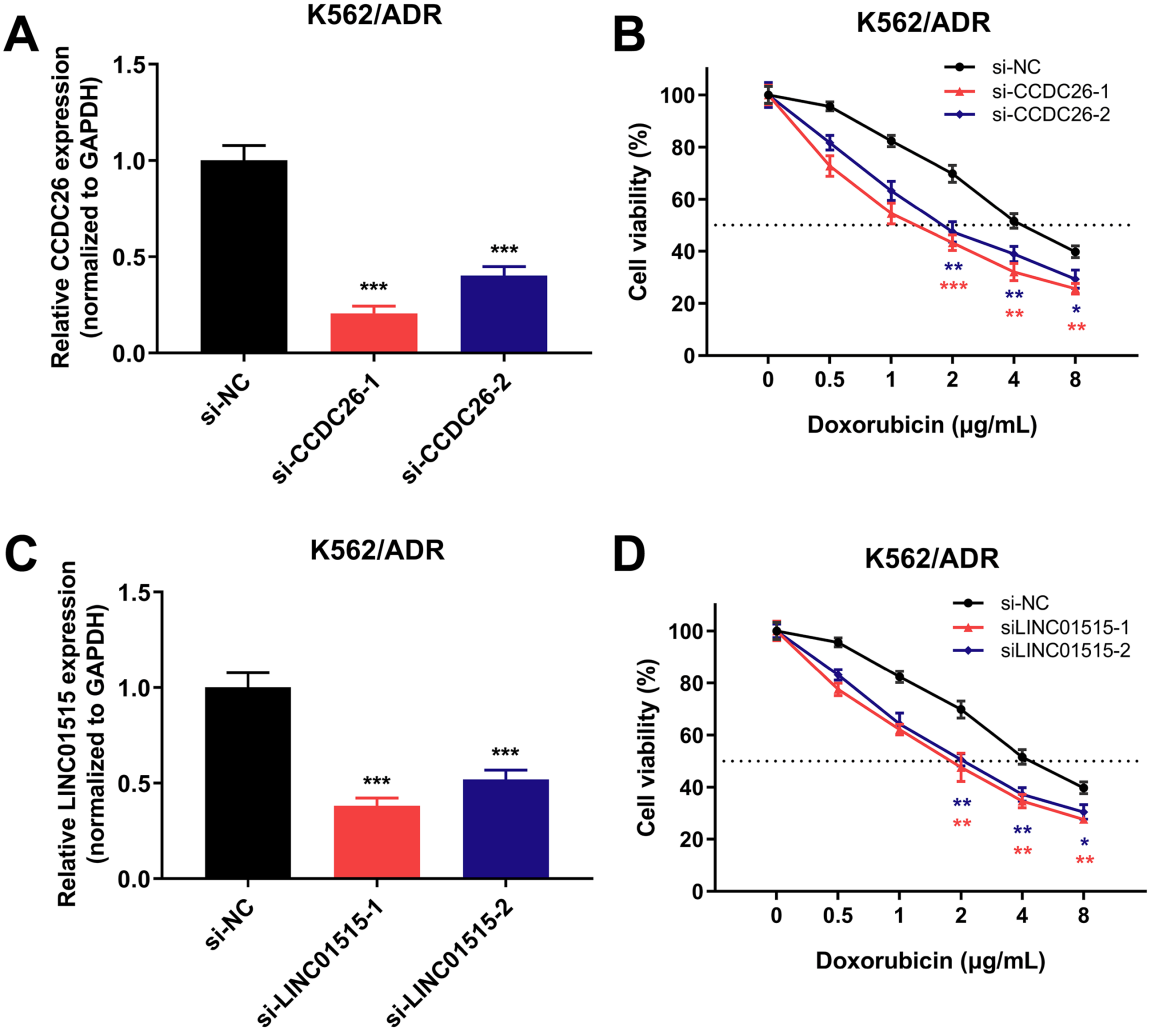
CCDC26 and LINC01515 expression in K562/ADR cells transfected with siRNAs and the cell survival rate after transfection. Forty-eight hours after transfection with si-NC, si-CCDC26 and si-LINC01515 in K562/ADR cells, CCDC26 (A) and LINC01515 (C) expression was detected by qRT-PCR. K562/ADR cells transfected with si-NC, si-CCDC26 and si-LINC01515 were treated with different concentrations of doxorubicin. The cell survival rate was evaluated by CCK-8 assay (B and D), and the OD value at wavelength of 450nm of K562/ADR cells that had been transfected with si-NC but not treated with doxorubicin was set to 100%. *P < 0.05, **P < 0.01 and ***P < 0.001 were statistically significant compared with the si-NC group.

To further investigate the role of lncRNAs in sensitivity of drug-resistant K562/ADR cells, three lncRNAs (CCDC26, LINC01515 and LINC01419) with the highest fold changes in our RNA-seq data were selected, and siRNAs were used to knock down the function of these lncRNAs. Using qRT-PCR, we found that 48 h later, the expression of CCDC26 and LINC01515 decreased compared with the negative control (si-NC) group (Figs. 7A and 7C). CCK-8 analysis showed that compared with the si-NC group (half-maximal inhibitory concentration (IC_50_) = 4.800 μg/mL), targeted inhibition of CCDC26 by siRNA-1 and siRNA-2 decreased the IC_50_ to 1.548 and 2.164 μg/mL, respectively. After knocking down LINC01515 by siRNA-1 and siRNA-2, the IC_50_ decreased to 1.951 and 2.283 μg/mL, respectively (Figs. 7B and 7D). These results indicated that knockdown of CCDC26 and LINC01515 could increase the sensitivity of K562/ADR cells to doxorubicin. However, decreasing the expression of LINC01419 did not affect the sensitivity of K562/ADR cells (Fig. S2).

### Knockdown of GLRX5 and DICER1 can enhance the sensitivity of drug-resistant K562/ADR cells

**Figure 8:**
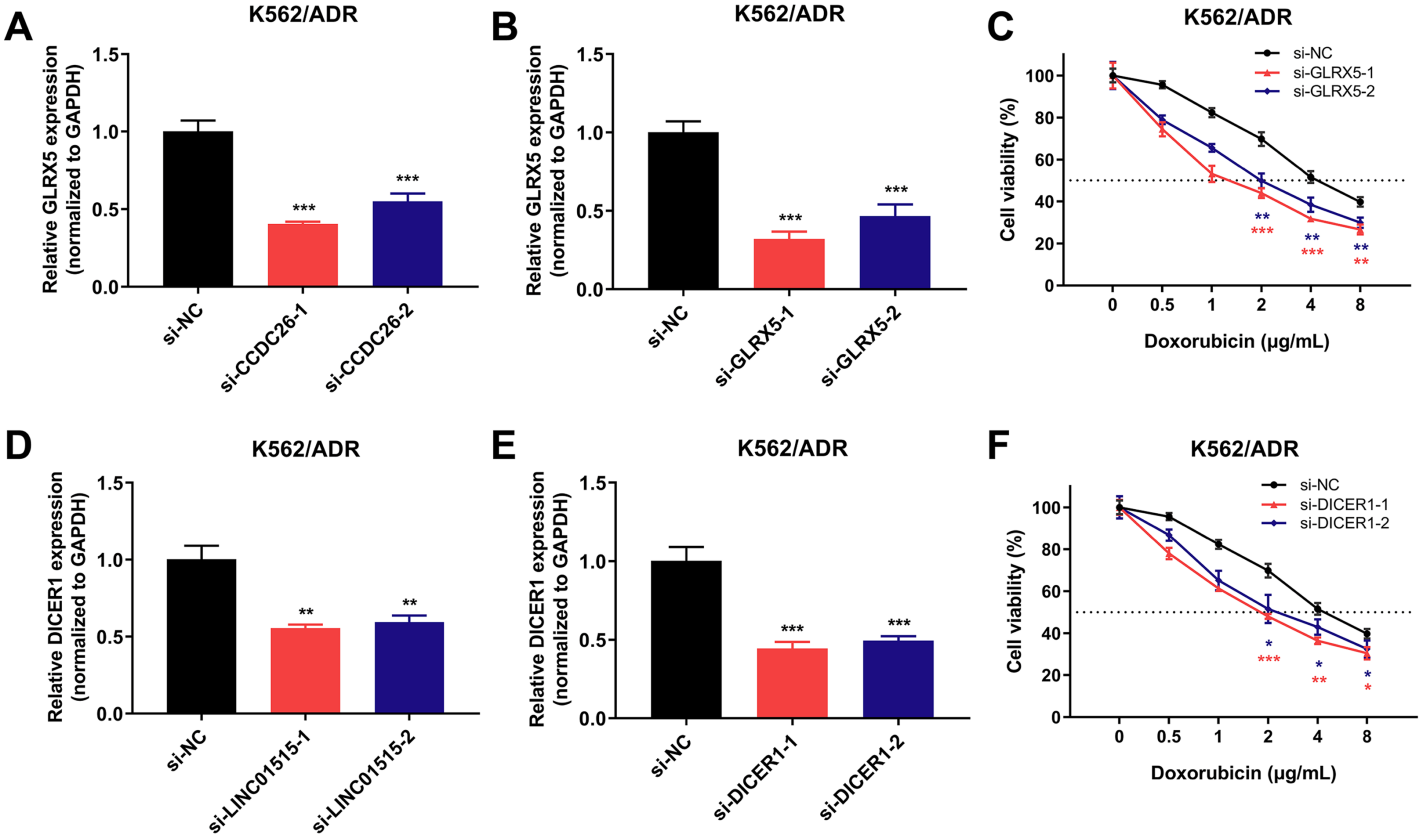
GLRX5 and DICER1 expression in K562/ADR cells transfected with siRNAs and the cell survival rate after transfection. Knockdown of CCDC26 and LINC01515 decreased the mRNA levels of GLRX5 (A) and DICER1 (D) in the drug-resistant K562/ADR cell line, respectively. Forty-eight hours after transfection with si-GLRX5 and si-DICER1 in K562/ADR cells, GLRX5 (B) and DICER1 (E) were downregulated, respectively. Knockdown of GLRX5 and DICER1 increased the sensitivity of K562/ADR cells to doxorubicin (C and F). *P < 0.05, **P < 0.01 and ***P < 0.001 were statistically significant compared with the si-NC group.

To dissect the possible regulatory mechanisms of CCDC26 and LINC01515 in drug resistance of CML, we separately extracted the ceRNA networks of them based on the constructed ceRNA networks shown in Fig. 5A. The results showed that CCDC26 and LINC01515 each have four potential ceRNA regulatory networks in CML sequencing (Fig. 5B). Interestingly, studies have reported that GLRX5 (target gene of CCDC26) and DICER1 (target gene of LINC01515) are associated with drug resistance (Lee et al., 2020; Wang et al., 2018a). In CML cell lines, we found that knocking down the expression of CCDC26 and LINC01515 can reduce the mRNA levels of GLRX5 (Fig. 8A) and DICER1 (Fig. 8D), respectively. CCK-8 analysis showed that compared with the si-NC group (IC_50_ = 4.800 μg/mL), targeted inhibition of GLRX5 by siRNA-1 and siRNA-2 (Fig. 8B) decreased the IC_50_ to 1.568 and 2.245 μg/mL, respectively (Fig. 8C). After knocking down DICER1 by siRNA-1 and siRNA-2 (Fig. 8E), the IC_50_ decreased to 2.036 and 2.609 μg/mL, respectively (Fig. 8F). These results indicated that knockdown of GLRX5 and DICER1 could increase the sensitivity of K562/ADR cells to doxorubicin. Therefore, combined with the results shown in Fig. 5B, CCDC26/miR-140-5p/GLRX5 and LINC01515/miR-425-5p/DICER1 may be potential ceRNA regulatory networks in drug resistance of CML.

## Discussion

MDR is related to the overexpression of ABC transporters, of which P-glycoprotein (encoded by ABCB1) is the most classical transporter. ABCB1 gene has been widely studied as a reliable biomarker of drug resistance. It is usually abnormally expressed in drug-resistant cancers (Cheng, Zhao & Liu, 2019; Zhou et al., 2019). Studies have reported that lncRNA is related to a variety of cellular biological processes of cancer, including proliferation, apoptosis, metastasis and invasion (Zhuang et al., 2019). Therefore, lncRNA has attracted wide attention as a potential biomarker for the diagnosis and prognosis of leukemia. A number of studies have shown that lncRNA is associated with chemotherapy resistance in leukemia (Bhat et al., 2020; Zebisch et al., 2016). For example, lncRNA TUG1 induces drug resistance to doxorubicin in AML through inhibiting the expression of miR-34a via EZH2 (Li, Song & Wang, 2019). On the contrary, lncRNA FENDRR downregulates MDR1 expression via sponging miR-184 and RNA binding protein HuR, thus reversing resistance of CML cells to doxorubicin (Zhang et al., 2019). But the underlying mechanism of MDR in K562/ADR cells caused by lncRNA is still poorly understood. Therefore, an in-depth understanding of lncRNA’s role in the progression and drug resistance of CML is essential for the discovery of novel prognostic markers and therapeutic targets and the development of reasonable and effective treatment strategies. In this study, we first reported the lncRNA expression profiles of K562/ADR cells, doxorubicin-resistant cells of CML, and further explored the possible lncRNA-miRNA-mRNA ceRNA mechanism of drug resistance by bioinformatics analysis.

To explore the biological mechanism of MDR in CML, we performed GO and KEGG enrichment analyses of targets of DElncRNAs between K562 and K562/ADR cells. As a result, GO enrichment analysis showed that the expression of DElncRNAs targets is closely related to metabolic pathway. Improving metabolic profile may play a positive role in the treatment of diseases (Chin et al., 2020). Studies have shown that drug resistance is closely related to metabolic changes. For example, in primary central nervous system lymphoma (CNS) derived cells, methotrexate (MTX) resistance is associated with changes of urea cycle and amino acid metabolism (Takashima, Hayano & Yamanaka, 2020). In addition, GO enrichment analysis showed that the expression of these genes is closely related to epigenetics. Hypermethylation of genes like BRCA1 and HOXA9 could potentially correlate with drug resistance (Pontikakis et al., 2017). Therefore, targets of DElncRNAs between K562 and K562/ADR cells may result in the MDR of CML through affecting metabolism and epigenetics. Our results of KEGG pathway enrichment analysis showed that DElncRNAs targets are mainly enriched in cancer-related pathways. Dihydromyricetin improves the sensitivity of doxorubicin-resistant cells via downregulating the expression of MDR1 and P-gp through the MAPK/ERK pathway (Sun et al., 2018). Similarly, EGCG reduces the expression of MDR1 and P-gp through the TFAP2A/VEGF pathway, thus restoring the sensitivity of gastric cancer cells to 5-fluorouracil (5-FU) (Tang et al., 2017). Above results suggest that MDR may be closely related to cell metabolism and cancer-related pathways, including MAPK and VEGF signaling pathways, which may help to overcome the MDR in CML. Therefore, these findings are of great significance to explore the pathogenesis and treatment of CML.

Some literatures have shown that ceRNA network participates in the occurrence of MDR in cancers, including nasopharyngeal carcinoma (Wang et al., 2020) and AML (Yu et al., 2020). However, except for one paper (Zhang et al., 2019), there is no other report on ceRNA regulatory network and its role in MDR-CML. What’s more, sequencing data is lacking to support studies on the ceRNA networks in MDR-CML. In order to explore the mechanism of the involvement of lncRNAs in MDR-CML, we constructed a ceRNA interaction network composed of 409 lncRNA-miRNA pairs and 306 miRNA-mRNA pairs based on 67 DElncRNAs, 58 DEmiRNAs and 192 DEmRNAs, which was visualized with Cytoscape software. Based on the sequencing results, we selected three lncRNAs with the highest expression levels (CCDC26, LINC01515 and LINC01419) for verification. The results showed that CCDC26, LINC01515 and LINC01419 were highly expressed in K562/ADR cells and inhibition of CCDC26 and LINC01515, but not inhibition of LINC01419, could enhance the sensitivity of K562/ADR cells to doxorubicin.

Previous studies have found that the most common copy number alterations in pediatric AML genome is an increase of burden in a region of the CCDC26 locus (Radtke et al., 2009). A new study on leukemia showed that CCDC26 is highly expressed in AML cells HL-60 and CML cells K562. CCDC26 promotes cell proliferation and invasion, and inhibits the apoptosis of HL-60 and K562 cells (Li et al., 2021). However, the biological function of LINC01515 has not been reported. LINC01419 can promote the cell proliferation and metastasis in gastric cancer, hepatocellular carcinoma, lung adenocarcinoma and osteosarcoma (Cheng et al., 2019; Dang et al., 2020; Gu et al., 2020; Wang, Zhang & Cui, 2019). Although LINC01419 is overexpressed in K562/ADR compared with K562 cell line, knockdown of LINC01419 does not increase the sensitivity of K562/ADR cells to doxorubicin. The reason may be that its high expression was caused by other molecules’ indirect regulation. It may not be an effector molecule directly regulating drug resistance. In this study, we first found that inhibition of CCDC26 and LINC01515 could enhance the sensitivity to doxorubicin in MDR-CML cells. GLRX5 and DICER1 are reported to be associated with drug resistance (Lee et al., 2020; Wang et al., 2018a). Silence of GLRX5 can reverse cisplatin resistance of head and neck cancer (HNC) cells through inducing ferroptosis (Lee et al., 2020). DICER1 involves in cisplatin resistance of epithelial ovarian cancer (EOC) cells through the miR-98-5p/DICER1/miR-152 pathway (Wang et al., 2018a). By using starBase v3.0 and miRcode database to predict the miRNAs that can be combined with CCDC26 and GLRX5, we found that miR-425-5p has the highest comprehensive score in the database; while miR-140-5p had the highest comprehensive score in the database among the miRNAs that could bind to LINC01515 and DICER1. In this study, we first found that CCDC26/miR-140-5p/GLRX5 and LINC01515/miR-425-5p/DICER1 may be potential ceRNA regulatory networks in the development and drug resistance of CML. CCDC26 and LINC01515 could positively regulate GLRX5 and DICER1, respectively. Inhibition of GLRX5 and DICER1 could enhance the sensitivity of K562/ADR cells to doxorubicin. In addition, as a member of ceRNA networks, miR-140-5p is involved in the chemotherapy resistance of cancers such as non-small cell lung cancer (NSCLC) (Fu et al., 2020), breast cancer (Zang et al., 2020) and hepatocellular carcinoma (HCC) (Fan et al., 2020). In breast cancer, miR-191 and miR-425 are highly expressed and promote the cell proliferation and metastasis by suppressing the expression of DICER1 (Zhang et al., 2018b). Previous studies have shown that high expression of miR-425-5p promotes cancer cells migration, invasion and proliferation. Knockout of miR-425-5p inhibits proliferation and migration of breast cancer cells (Xiao et al., 2019). PWRN1 inhibits cell proliferation and metastasis through the p53 signaling pathway as a ceRNA targeting miR-425-5p, thus inhibiting the development of gastric cancer (Chen et al., 2018). It is worth noting that miR-425-5p promotes the chemoresistance of several cancer cells, including 5-FU and oxaliplatin (OX)-resistant colorectal cancer cells and cisplatin-resistant laryngeal cancer cells (Jin et al., 2019; Ma et al., 2019a). Therefore, we postulated that CCDC26/miR-140-5p/GLRX5 and LINC01515/miR-425-5p/DICER1 may be potential ceRNA regulatory networks in disease development and drug resistance.

As far as we know, this is the first report to construct and analyze the ceRNA network of lncRNAs, miRNAs and mRNAs related to chemotherapeutic drug or targeted drug resistance in K562/ADR cells. What needs to be admitted is that our research has some limitations. For example, the specific roles of miR-140-5p and miR-425-5p in drug resistance of CML have not been verified in K562/ADR cells, and clear ceRNA mechanism has not been confirmed by RNA immunoprecipitation (RIP) or luciferase gene reporter assays. Further studies are still needed to clarify other biological functions of CCDC26, LINC01515, GLRX5 and DICER1 in CML.

## Conclusions

In summary, we have constructed a ceRNA network, which may be linked to MDR in CML. Furthermore, we found that CCDC26/miR-140-5p/GLRX5 and LINC01515/miR-425-5p/DICER1 may be potential ceRNA regulatory networks in the development and drug resistance of CML. LncRNA-miRNA-mRNA ceRNA regulatory network is promising to act as a therapeutic target of CML disease development and drug resistance. These results will foster our insights into the ceRNA mechanism of leukemia drug resistance.

## Supplemental Information

10.7717/peerj.11429/supp-1 Supplemental Information 1 GO and KEGG analyses of DElncRNA target genes between K562/ADR and K562 cells.

10.7717/peerj.11429/supp-2 Supplemental Information 2 The top 10 upregulated and downregulated DElncRNAs.

10.7717/peerj.11429/supp-3 Supplemental Information 3 The top 20 upregulated and downregulated DEmRNAs.

10.7717/peerj.11429/supp-4 Supplemental Information 4 qRT-PCR was used to validate the expression of mRNAs in the drug-resistant K562/ADR cell line. All data are represented as mean ± SD for at least three independent experiments. *P < 0.05, **P < 0.01 and ***P < 0.001 were statistically significant compared with the control group.

10.7717/peerj.11429/supp-5 Supplemental Information 5 LINC01419 expression in K562/ADR cells transfected with siRNAs and the cell survival rate after transfection. 48 hours after transfection with siRNAs in K562/ADR cells, LINC01419 expression was downregulated (A). ****Knockdown of LINC01419 did not affect the sensitivity of K562/ADR cells to doxorubicin (B). *P < 0.05, **P < 0.01 and ***P < 0.001 were statistically significant compared with the si-NC group.

10.7717/peerj.11429/supp-6 Supplemental Information 6 qRT-PCR was used to validate the expression of lncRNAs and mRNAs in the drug-resistant K562/ADR cell line. Raw data on the expression of lncRNAs and mRNAs in the drug-resistant K562/ADR cell line; these data were applied in data analysis and preparation of Figs. 3 and S1.

10.7717/peerj.11429/supp-7 Supplemental Information 7 qRT-PCR was used to detect the expression of CCDC26, LINC01515, LINC01419, GLRX5 and DICER1 in K562/ADR cells transfected with siRNAs. Raw data on the expression of CCDC26, LINC01515, LINC01419, GLRX5 and DICER1 in K562/ADR cells transfected with siRNAs; these data were applied in data analysis and preparation of Figs. 7, 8 and S2.

10.7717/peerj.11429/supp-8 Supplemental Information 8 The cell survival rate of K562/ADR cells transfected with siRNAs. These data were applied in data analysis and preparation of Figs. 7, 8 and S2.

## Additional Information and Declarations

### DNA Deposition

The following information was supplied regarding the deposition of DNA sequences: The CCDC26 and LINC01515 sequences are available at GenBank: NR_130917 and NR_120647.

### Data Availability

The following information was supplied regarding data availability: The *Homo sapiens* LncRNA Raw sequence reads are available at NCBI SRA: PRJNA648744. The raw data are available in the Supplemental Files.

## References

[ref-1] An X, Tiwari AK, Sun Y, Ding PR, Ashby CR, Chen ZS (2010). BCR-ABL tyrosine kinase inhibitors in the treatment of Philadelphia chromosome positive chronic myeloid leukemia: a review. Leukemia Research.

[ref-2] Assaraf YG, Brozovic A, Gonçalves AC, Jurkovicova D, Linē A, Machuqueiro M, Saponara S, Sarmento-Ribeiro AB, Xavier CPR, Vasconcelos MH (2019). The multi-factorial nature of clinical multidrug resistance in cancer. Drug Resistance Updates.

[ref-3] Bhat AA, Younes SN, Raza SS, Zarif L, Nisar S, Ahmed I, Mir R, Kumar S, Sharawat SK, Hashem S, Elfaki I, Kulinski M, Kuttikrishnan S, Prabhu KS, Khan AQ, Yadav SK, El-Rifai W, Zargar MA, Zayed H, Haris M, Uddin S (2020). Role of non-coding RNA networks in leukemia progression, metastasis and drug resistance. Molecular Cancer.

[ref-4] Chen Z, Ju H, Yu S, Zhao T, Jing X, Li P, Jia J, Li N, Tan B, Li Y (2018). Prader-Willi region non-protein coding RNA 1 suppressed gastric cancer growth as a competing endogenous RNA of miR-425-5p. Clinical Science.

[ref-5] Cheng FH, Zhao ZS, Liu WD (2019). Long non-coding RNA ROR regulated ABCB1 to induce cisplatin resistance in osteosarcoma by sponging miR-153-3p. European Review for Medical and Pharmacological Sciences.

[ref-6] Cheng Z, Hou S, Wu Y, Wang X, Sun Y, Liu B, Yuan M (2019). LINC01419 promotes cell proliferation and metastasis in lung adenocarcinoma via sponging miR-519b-3p to up-regulate RCCD1. Biochemical and Biophysical Research Communications.

[ref-7] Chin KY, Wong SK, Ekeuku SO, Pang KL (2020). Relationship between metabolic syndrome and bone health: an evaluation of epidemiological studies and mechanisms involved. Diabetes, Metabolic Syndrome and Obesity: Targets and Therapy.

[ref-9] Dang H, Chen L, Tang P, Cai X, Zhang W, Zhang R, Huang A, Tang H (2020). LINC01419 promotes cell proliferation and metastasis in hepatocellular carcinoma by enhancing NDRG1 promoter activity. Cellular Oncology.

[ref-10] de Lavallade H, Apperley JF, Khorashad JS, Milojkovic D, Reid AG, Bua M, Szydlo R, Olavarria E, Kaeda J, Goldman JM, Marin D (2008). Imatinib for newly diagnosed patients with chronic myeloid leukemia: incidence of sustained responses in an intention-to-treat analysis. Journal of Clinical Oncology.

[ref-11] Fan L, Huang X, Chen J, Zhang K, Gu YH, Sun J, Cui SY (2020). Long noncoding RNA MALAT1 contributes to sorafenib resistance by targeting miR-140-5p/aurora-a signaling in hepatocellular carcinoma. Molecular Cancer Therapeutics.

[ref-12] Fu J, Cai H, Wu Y, Fang S, Wang D (2020). Elevation of FGD5-AS1 contributes to cell progression by improving cisplatin resistance against non-small cell lung cancer cells through regulating miR-140-5p/WEE1 axis. Gene.

[ref-13] Genovese I, Ilari A, Assaraf YG, Fazi F, Colotti G (2017). Not only P-glycoprotein: amplification of the ABCB1-containing chromosome region 7q21 confers multidrug resistance upon cancer cells by coordinated overexpression of an assortment of resistance-related proteins. Drug Resistance Updates.

[ref-14] Gu Z, Wu S, Wang J, Zhao S (2020). Long non-coding RNA LINC01419 mediates miR-519a-3p/PDRG1 axis to promote cell progression in osteosarcoma. Cancer Cell International.

[ref-15] Gurney H, Wong M, Balleine RL, Rivory LP, McLachlan AJ, Hoskins JM, Wilcken N, Clarke CL, Mann GJ, Collins M, Delforce SE, Lynch K, Schran H (2007). Imatinib disposition and ABCB1 (MDR1, P-glycoprotein) genotype. Clinical Pharmacology & Therapeutics.

[ref-17] Huarte M (2015). The emerging role of lncRNAs in cancer. Nature Medicine.

[ref-18] Jabbour E, Kantarjian H (2020). Chronic myeloid leukemia: 2020 update on diagnosis, therapy and monitoring. American Journal of Hematology.

[ref-19] Jin G, Liu Y, Zhang J, Bian Z, Yao S, Fei B, Zhou L, Yin Y, Huang Z (2019). A panel of serum exosomal microRNAs as predictive markers for chemoresistance in advanced colorectal cancer. Cancer Chemotherapy and Pharmacology.

[ref-20] Kong X, Hu S, Yuan Y, Du Y, Zhu Z, Song Z, Lu S, Zhao C, Yan D (2020). Analysis of lncRNA, miRNA and mRNA-associated ceRNA networks and identification of potential drug targets for drug-resistant non-small cell lung cancer. Journal of Cancer.

[ref-21] Le NQK, Yapp EKY, Nagasundaram N, Chua MCH, Yeh HY (2019). Computational identification of vesicular transport proteins from sequences using deep gated recurrent units architecture. Computational and Structural Biotechnology Journal.

[ref-22] Le NQK, Yapp EKY, Yeh HY (2019). ET-GRU: using multi-layer gated recurrent units to identify electron transport proteins. BMC Bioinformatics.

[ref-23] Lee J, You JH, Shin D, Roh JL (2020). Inhibition of Glutaredoxin 5 predisposes cisplatin-resistant head and neck cancer cells to ferroptosis. Theranostics.

[ref-24] Li C, Mu J, Shi Y, Xin H (2021). LncRNA CCDC26 Interacts with CELF2 protein to enhance myeloid leukemia cell proliferation and invasion via the circRNA_ANKIB1/miR-195-5p/PRR11 Axis. Cell Transplantation.

[ref-25] Li Q, Song W, Wang J (2019). TUG1 confers Adriamycin resistance in acute myeloid leukemia by epigenetically suppressing miR-34a expression via EZH2. Biomedicine & Pharmacotherapy.

[ref-26] Liu H, Wang S, Zhou S, Meng Q, Ma X, Song X, Wang L, Jiang W (2019). Drug resistance-related competing interactions of lncRNA and mRNA across 19 cancer types. Molecular Therapy - Nucleic Acids.

[ref-27] Liu J, Li Y, Tong J, Gao J, Guo Q, Zhang L, Wang B, Zhao H, Wang H, Jiang E, Kurita R, Nakamura Y, Tanabe O, Engel JD, Bresnick EH, Zhou J, Shi L (2018). Long non-coding RNA-dependent mechanism to regulate heme biosynthesis and erythrocyte development. Nature Communications.

[ref-28] Liu Z, Wang Y, Yuan S, Wen F, Liu J, Zou L, Zhang J (2021). Regulatory role of long non-coding RNA UCA1 in signaling pathways and its clinical applications. Oncology Letters.

[ref-29] Lu Y, Zhao X, Liu Q, Li C, Graves-Deal R, Cao Z, Singh B, Franklin JL, Wang J, Hu H, Wei T, Yang M, Yeatman TJ, Lee E, Saito-Diaz K, Hinger S, Patton JG, Chung CH, Emmrich S, Klusmann JH, Fan D, Coffey RJ (2017). lncRNA MIR100HG-derived miR-100 and miR-125b mediate cetuximab resistance via Wnt/β-catenin signaling. Nature Medicine.

[ref-30] Ma Y, Yuwen D, Chen J, Zheng B, Gao J, Fan M, Xue W, Wang Y, Li W, Shu Y, Xu Q, Shen Y (2019a). Exosomal transfer of cisplatin-induced miR-425-3p confers cisplatin resistance in NSCLC through activating autophagy. International Journal of Nanomedicine.

[ref-31] Ma Z, Zhang J, Xu X, Qu Y, Dong H, Dang J, Huo Z, Xu G (2019b). LncRNA expression profile during autophagy and Malat1 function in macrophages. PLOS ONE.

[ref-32] Pontikakis S, Papadaki C, Tzardi M, Trypaki M, Sfakianaki M, Koinis F, Lagoudaki E, Giannikaki L, Kalykaki A, Kontopodis E, Saridaki Z, Malamos N, Georgoulias V, Souglakos J (2017). Predictive value of ATP7b, BRCA1, BRCA2, PARP1, UIMC1 (RAP80), HOXA9, DAXX, TXN (TRX1), THBS1 (TSP1) and PRR13 (TXR1) genes in patients with epithelial ovarian cancer who received platinum-taxane first-line therapy. The Pharmacogenomics Journal.

[ref-33] Qi M, Yu B, Yu H, Li F (2020). Integrated analysis of a ceRNA network reveals potential prognostic lncRNAs in gastric cancer. Cancer Medicine.

[ref-34] Radtke I, Mullighan CG, Ishii M, Su X, Cheng J, Ma J, Ganti R, Cai Z, Goorha S, Pounds SB, Cao X, Obert C, Armstrong J, Zhang J, Song G, Ribeiro RC, Rubnitz JE, Raimondi SC, Shurtleff SA, Downing JR (2009). Genomic analysis reveals few genetic alterations in pediatric acute myeloid leukemia. Proceedings of the National Academy of Sciences of the United States of America.

[ref-35] Rumjanek VM, Vidal RS, Maia RC (2013). Multidrug resistance in chronic myeloid leukaemia: how much can we learn from MDR-CML cell lines?. Bioscience Reports.

[ref-36] Smallegan MJ, Rinn JL (2019). Linking long noncoding RNA to drug resistance. Proceedings of the National Academy of Sciences of the United States of America.

[ref-37] Strati P, Kantarjian H, Thomas D, O’Brien S, Konoplev S, Jorgensen JL, Luthra R, Abruzzo L, Jabbour E, Quintas-Cardama A, Borthakur G, Faderl S, Ravandi F, Cortes J (2014). HCVAD plus imatinib or dasatinib in lymphoid blastic phase chronic myeloid leukemia. Cancer.

[ref-38] Sun Y, Wang C, Meng Q, Liu Z, Huo X, Sun P, Sun H, Ma X, Peng J, Liu K (2018). Targeting P-glycoprotein and SORCIN: Dihydromyricetin strengthens anti-proliferative efficiency of adriamycin via MAPK/ERK and Ca(2+) -mediated apoptosis pathways in MCF-7/ADR and K562/ADR. Journal of Cellular Physiology.

[ref-39] Takashima Y, Hayano A, Yamanaka R (2020). Metabolome analysis reveals excessive glycolysis via PI3K/AKT/mTOR and RAS/MAPK signaling in methotrexate-resistant primary CNS lymphoma-derived cells. Clinical Cancer Research.

[ref-40] Tang H, Zeng L, Wang J, Zhang X, Ruan Q, Wang J, Cui S, Yang D (2017). Reversal of 5-fluorouracil resistance by EGCG is mediate by inactivation of TFAP2A/VEGF signaling pathway and down-regulation of MDR-1 and P-gp expression in gastric cancer. Oncotarget.

[ref-41] Wang LL, Zhang L, Cui XF (2019). Downregulation of long noncoding RNA LINC01419 inhibits cell migration, invasion, and tumor growth and promotes autophagy via inactivation of the PI3K/Akt1/mTOR pathway in gastric cancer. Therapeutic Advances in Medical Oncology.

[ref-42] Wang W, Lou W, Ding B, Yang B, Lu H, Kong Q, Fan W (2019). A novel mRNA-miRNA-lncRNA competing endogenous RNA triple sub-network associated with prognosis of pancreatic cancer. Aging (Albany NY).

[ref-43] Wang X, Wang C, Xu H, Xie H (2020). Long non-coding RNA SLC25A21-AS1 promotes multidrug resistance in nasopharyngeal carcinoma by regulating miR-324-3p/IL-6 Axis. Cancer Management and Research.

[ref-44] Wang Y, Bao W, Liu Y, Wang S, Xu S, Li X, Li Y, Wu S (2018a). miR-98-5p contributes to cisplatin resistance in epithelial ovarian cancer by suppressing miR-152 biogenesis via targeting Dicer1. Cell Death & Disease.

[ref-45] Wang Y, Zong S, Wu L, Zhang Y, Wang Z, Wang Z, Chen B, Cui Y (2018b). Evaluation of multidrug resistance of leukemia using surface-enhanced raman scattering method for clinical applications. ACS Applied Materials & Interfaces.

[ref-46] Wu Q, Yang Z, Nie Y, Shi Y, Fan D (2014). Multi-drug resistance in cancer chemotherapeutics: mechanisms and lab approaches. Cancer Letters.

[ref-47] Xiao S, Zhu H, Luo J, Wu Z, Xie M (2019). miR‐425‐5p is associated with poor prognosis in patients with breast cancer and promotes cancer cell progression by targeting PTEN. Oncology Reports.

[ref-48] Xiao Y, Jiao C, Lin Y, Chen M, Zhang J, Wang J, Zhang Z (2017). lncRNA UCA1 contributes to imatinib resistance by acting as a ceRNA against miR-16 in chronic myeloid leukemia cells. Dna and Cell Biology.

[ref-51] Yi YJ, Jia XH, Wang JY, Li YJ, Wang H, Xie SY (2016). Knockdown of HOXA10 reverses the multidrug resistance of human chronic mylogenous leukemia K562/ADM cells by downregulating P-gp and MRP-1. International Journal of Molecular Medicine.

[ref-52] Yu Y, Kou D, Liu B, Huang Y, Li S, Qi Y, Guo Y, Huang T, Qi X, Jia L (2020). LncRNA MEG3 contributes to drug resistance in acute myeloid leukemia by positively regulating ALG9 through sponging miR-155. International Journal of Laboratory Hematology.

[ref-53] Zang H, Li Y, Zhang X, Huang G (2020). Circ-RNF111 contributes to paclitaxel resistance in breast cancer by elevating E2F3 expression via miR-140-5p. Thoracic Cancer.

[ref-54] Zebisch A, Hatzl S, Pichler M, Wölfler A, Sill H (2016). Therapeutic resistance in acute myeloid leukemia: the role of non-coding RNAs. International Journal of Molecular Sciences.

[ref-55] Zhang F, Ni H, Li X, Liu H, Xi T, Zheng L (2019). LncRNA FENDRR attenuates adriamycin resistance via suppressing MDR1 expression through sponging HuR and miR-184 in chronic myelogenous leukaemia cells. Febs Letters.

[ref-56] Zhang JY, Lin MT, Yi T, Tang YN, Fan LL, He XC, Zhao ZZ, Chen HB (2013). Apoptosis sensitization by Euphorbia factor L1 in ABCB1-mediated multidrug resistant K562/ADR cells. Molecules.

[ref-57] Zhang W, Yuan W, Song J, Wang S, Gu X (2018a). LncRNA CPS1-IT1 suppresses EMT and metastasis of colorectal cancer by inhibiting hypoxia-induced autophagy through inactivation of HIF-1alpha. Biochimie.

[ref-58] Zhang X, Wu M, Chong QY, Zhang W, Qian P, Yan H, Qian W, Zhang M, Lobie PE, Zhu T (2018b). Amplification of hsa-miR-191/425 locus promotes breast cancer proliferation and metastasis by targeting DICER1. Carcinogenesis.

[ref-59] Zhou HH, Chen X, Cai LY, Nan XW, Chen JH, Chen XX, Yang Y, Xing ZH, Wei MN, Li Y, Wang ST, Liu K, Shi Z, Yan XJ (2019). Erastin reverses ABCB1-mediated docetaxel resistance in ovarian cancer. Frontiers in Oncology.

[ref-60] Zhuang C, Ma Q, Zhuang C, Ye J, Zhang F, Gui Y (2019). LncRNA GClnc1 promotes proliferation and invasion of bladder cancer through activation of MYC. FASEB Journal.

