# Supplementary material for: Analysis of ceRNA networks and identification of potential drug targets for drug-resistant leukemia cell K562/ADR
Source: PeerJ. 2021 May 25;9:e11429. doi: 10.7717/peerj.11429 (PMC8162247; doi:10.7717/peerj.11429)
Supplement: Supplemental Information 2 [file peerj-09-11429-s002.docx]

**Table S2** **The top 10 upregulated and downregulated DElncRNAs.**

| Transcript_id | Gene_id | Gene_name | K562_ADR_FPKM | K562_FPKM | Log2(foldchange) | *P*-value | *q*-value | Regulation |
| --- | --- | --- | --- | --- | --- | --- | --- | --- |
| ENST00000522365.1 | ENSG00000253898.1 | LINC01419 | 2.601561333 | 0.015914333 | 7.352907188 | 0.000722582 | 0.016025264 | Up |
| ENST00000647094.1 | ENSG00000229140.9 | CCDC26 | 12.11627867 | 0.074728333 | 7.341075603 | 0.000361249 | 0.01154224 | Up |
| ENST00000433152.8 | ENSG00000228065.10 | LINC01515 | 5.654575667 | 0.043149333 | 7.033936685 | 0.000224691 | 0.009656331 | Up |
| ENST00000649415 | ENSG00000285751 | AC021723.2 | 5.774208 | 0.051480667 | 6.809448531 | 0.002285437 | 0.029858616 | Up |
| ENST00000582086.2 | ENSG00000263745.6 | AP005230.1 | 4.402196333 | 0.061241333 | 6.167573986 | 0.001231674 | 0.020818851 | Up |
| ENST00000560195.1 | ENSG00000259611.1 | LINC01582 | 5.009733333 | 0.098694 | 5.665627621 | 0.000730784 | 0.01610022 | Up |
| ENST00000433446.1 | ENSG00000224046.1 | AC005076.5 | 101.252856 | 2.176043 | 5.540111726 | 0.000263239 | 0.010097372 | Up |
| ENST00000561473.1 | ENSG00000261462.1 | DMTF1 | 18.904693 | 0.482231333 | 5.292875217 | 0.004148054 | 0.041815735 | Up |
| ENST00000416560.5 | ENSG00000182165.17 | TP53TG1 | 57.41923133 | 1.728325333 | 5.054087302 | 7.56E-05 | 0.006589236 | Up |
| ENST00000414790.6 | ENSG00000130600.18 | H19 | 10.966323 | 0.395042 | 4.794930016 | 0.000233736 | 0.00978751 | Up |
| ENST00000623095.3 | ENSG00000277067.4 | CRYAA2 | 0.017832667 | 12.912141 | -9.499990065 | 8.65E-06 | 0.002881694 | Down |
| ENST00000594318.1 | ENSG00000267886.1 | ZNF730 | 0.015030333 | 2.680411 | -7.478433418 | 0.001743574 | 0.025842867 | Down |
| ENST00000560097.1 | ENSG00000259692.5 | MEX3B | 0.046835333 | 2.189242333 | -5.546690516 | 0.002553518 | 0.031821562 | Down |
| ENST00000598754.1 | ENSG00000268621.5 | IGFL2-AS1 | 0.063864 | 2.695994333 | -5.399670738 | 0.000943655 | 0.018383019 | Down |
| ENST00000445293.6 | ENSG00000232053.6 | STMP1 | 0.180008333 | 6.070608 | -5.075705415 | 0.000228584 | 0.009686819 | Down |
| ENST00000585816.1 | ENSG00000267383.6 | ZNF682 | 0.084909 | 2.764818333 | -5.025123397 | 0.000270962 | 0.010172978 | Down |
| ENST00000649506 | ENSG00000241743 | XACT | 0.133628333 | 3.959805333 | -4.889131667 | 0.001528248 | 0.023814487 | Down |
| ENST00000510284.1 | ENSG00000251230.5 | MIR3945HG | 0.190540667 | 3.938775333 | -4.369576282 | 0.000441484 | 0.012554359 | Down |
| ENST00000634373.1 | ENSG00000283095.1 | PPP2R2D | 0.136144667 | 2.181265333 | -4.0019529 | 0.000174714 | 0.008798815 | Down |
| ENST00000648102 | ENSG00000285774 | AL133444.1 | 0.573619667 | 8.392687 | -3.870966384 | 0.000247883 | 0.009915549 | Down |

**Notes.** DE, differentially expressed; FPKM, Fragments Per Kilobase of transcript per Million Fragments.
