# Supplementary material for: Analysis of ceRNA networks and identification of potential drug targets for drug-resistant leukemia cell K562/ADR
Source: PeerJ. 2021 May 25;9:e11429. doi: 10.7717/peerj.11429 (PMC8162247; doi:10.7717/peerj.11429)
Supplement: Supplemental Information 3 [file peerj-09-11429-s003.docx]

**Table S3 The top 20 upregulated and downregulated DEmRNAs.**

| Transcript_id | Gene_id | Gene_name | K562_ADR_FPKM | K562_FPKM | Log2(foldchange) | *P*-value | *q*-value | Regulation |
| --- | --- | --- | --- | --- | --- | --- | --- | --- |
| ENST00000425642 | ENSG00000171130 | ATP6V0E2 | 1.825972 | 0.002643 | 9.432272502 | 0.000138021 | 0.007998082 | Up |
| ENST00000622132 | ENSG00000085563 | ABCB1 | 1251.936849 | 2.418843 | 9.015628944 | 1.19E-05 | 0.002881694 | Up |
| ENST00000419147 | ENSG00000005469 | CROT | 12.41114267 | 0.024185667 | 9.003267926 | 0.001002342 | 0.018768883 | Up |
| ENST00000262650 | ENSG00000078747 | ITCH | 5.470838333 | 0.016874333 | 8.340787608 | 0.000205179 | 0.009378739 | Up |
| ENST00000407818 | ENSG00000068796 | KIF2A | 12.07827933 | 0.039542667 | 8.254789062 | 0.004286003 | 0.042669597 | Up |
| ENST00000536003 | ENSG00000175575 | PAAF1 | 8.674002667 | 0.029960333 | 8.177500401 | 1.27E-05 | 0.002881694 | Up |
| ENST00000310954 | ENSG00000173930 | SLCO4C1 | 3.475654667 | 0.012438333 | 8.126347751 | 0.00033367 | 0.011206939 | Up |
| ENST00000380338 | ENSG00000171843 | MLLT3 | 2.285346667 | 0.008267667 | 8.110717087 | 6.17E-05 | 0.006013011 | Up |
| ENST00000455575 | ENSG00000135185 | TMEM243 | 12.82437433 | 0.051709333 | 7.954248022 | 0.000279704 | 0.010280295 | Up |
| ENST00000285379 | ENSG00000104267 | CA2 | 15.73633333 | 0.073671667 | 7.738773827 | 0.000250489 | 0.009944849 | Up |
| ENST00000535549 | ENSG00000134954 | ETS1 | 10.208272 | 0.059609333 | 7.419984722 | 0.000416794 | 0.012334918 | Up |
| ENST00000264360 | ENSG00000138650 | PCDH10 | 2.979558333 | 0.017515 | 7.410363694 | 0.000112192 | 0.007355565 | Up |
| ENST00000314589 | ENSG00000187098 | MITF | 2.719310333 | 0.017226667 | 7.302453425 | 0.00114197 | 0.020112114 | Up |
| ENST00000346874 | ENSG00000148498 | PARD3 | 1.786075 | 0.011814 | 7.240151335 | 0.001898661 | 0.026851642 | Up |
| ENST00000301396 | ENSG00000141456 | PELP1 | 7.663162667 | 0.052633 | 7.185828491 | 0.001547886 | 0.024033042 | Up |
| ENST00000536354 | ENSG00000151790 | TDO2 | 15.99883967 | 0.119482333 | 7.065026149 | 0.002363538 | 0.030479972 | Up |
| ENST00000412504 | ENSG00000196083 | IL1RAP | 4.736187333 | 0.038745333 | 6.93355978 | 9.38E-06 | 0.002881694 | Up |
| ENST00000493412 | ENSG00000198947 | DMD | 2.713699333 | 0.024779667 | 6.774960294 | 0.000166959 | 0.008693457 | Up |
| ENST00000336904 | ENSG00000145335 | SNCA | 3.294557 | 0.035639333 | 6.530470327 | 9.14E-05 | 0.007169599 | Up |
| ENST00000228425 | ENSG00000110841 | PPFIBP1 | 7.177554 | 0.077727 | 6.528932635 | 0.002799514 | 0.033161256 | Up |
| ENST00000468410 | ENSG00000105856 | HBP1 | 0.001171333 | 4.869588333 | -12.02143241 | 0.000549597 | 0.013789062 | Down |
| ENST00000302804 | ENSG00000105146 | AURKC | 0.001589667 | 2.688930667 | -10.72409256 | 0.000256182 | 0.010048144 | Down |
| ENST00000375754 | ENSG00000165025 | SYK | 0.010767667 | 12.61154433 | -10.19382358 | 7.40E-06 | 0.002870183 | Down |
| ENST00000404739 | ENSG00000076685 | NT5C2 | 0.010179667 | 9.549089 | -9.873528973 | 6.55E-07 | 0.001726574 | Down |
| ENST00000265087 | ENSG00000113739 | STC2 | 0.008602 | 7.523907 | -9.77259417 | 1.65E-05 | 0.003360375 | Down |
| ENST00000421093 | ENSG00000165804 | ZNF219 | 0.004524333 | 2.252938333 | -8.959886885 | 0.000137402 | 0.007998082 | Down |
| ENST00000319675 | ENSG00000176566 | DCAF4L2 | 0.030541333 | 13.06151467 | -8.740343445 | 2.17E-05 | 0.00372118 | Down |
| ENST00000531792 | ENSG00000165325 | DEUP1 | 0.013407333 | 5.676605 | -8.725862227 | 0.001417719 | 0.022726517 | Down |
| ENST00000369429 | ENSG00000092607 | TBX15 | 0.011390333 | 4.33462 | -8.571951746 | 8.54E-05 | 0.006968739 | Down |
| ENST00000606017 | ENSG00000272398 | CD24 | 0.03076 | 10.979866 | -8.479589229 | 0.00234219 | 0.030269862 | Down |
| ENST00000317802 | ENSG00000178021 | TSPYL6 | 0.033795 | 8.508196 | -7.975899644 | 4.08E-05 | 0.0049531 | Down |
| ENST00000316950 | ENSG00000196437 | ZNF569 | 0.012403 | 2.690522333 | -7.761053355 | 0.00195064 | 0.027248284 | Down |
| ENST00000054950 | ENSG00000049449 | RCN1 | 0.037496 | 6.891611333 | -7.52196083 | 0.000111915 | 0.007355565 | Down |
| ENST00000305747 | ENSG00000172292 | CERS6 | 0.027959 | 5.101544667 | -7.511477567 | 9.61E-07 | 0.001726574 | Down |
| ENST00000274364 | ENSG00000145703 | IQGAP2 | 0.038379333 | 6.456513 | -7.39428175 | 1.58E-06 | 0.002185775 | Down |
| ENST00000247470 | ENSG00000103490 | PYCARD | 0.032296667 | 4.864051667 | -7.234629471 | 0.000351189 | 0.011428921 | Down |
| ENST00000374391 | ENSG00000012779 | ALOX5 | 0.012798333 | 1.89409 | -7.209405127 | 0.000944529 | 0.018383019 | Down |
| ENST00000409599 | ENSG00000115604 | IL18R1 | 0.014993667 | 2.034025667 | -7.08384084 | 0.000251887 | 0.009944849 | Down |
| ENST00000381089 | ENSG00000169059 | VCX3A | 0.056900333 | 7.009078333 | -6.944643834 | 6.17E-06 | 0.002756755 | Down |
| ENST00000394957 | ENSG00000107738 | VSIR | 0.052153333 | 6.036393333 | -6.854783541 | 4.47E-05 | 0.005248112 | Down |

**Notes.** DE, differentially expressed; FPKM, Fragments Per Kilobase of transcript per Million Fragments.
