# Supplementary figures and images for: Analysis of ceRNA networks and identification of potential drug targets for drug-resistant leukemia cell K562/ADR
Source: PeerJ. 2021 May 25;9:e11429. doi: 10.7717/peerj.11429 (PMC8162247; doi:10.7717/peerj.11429)

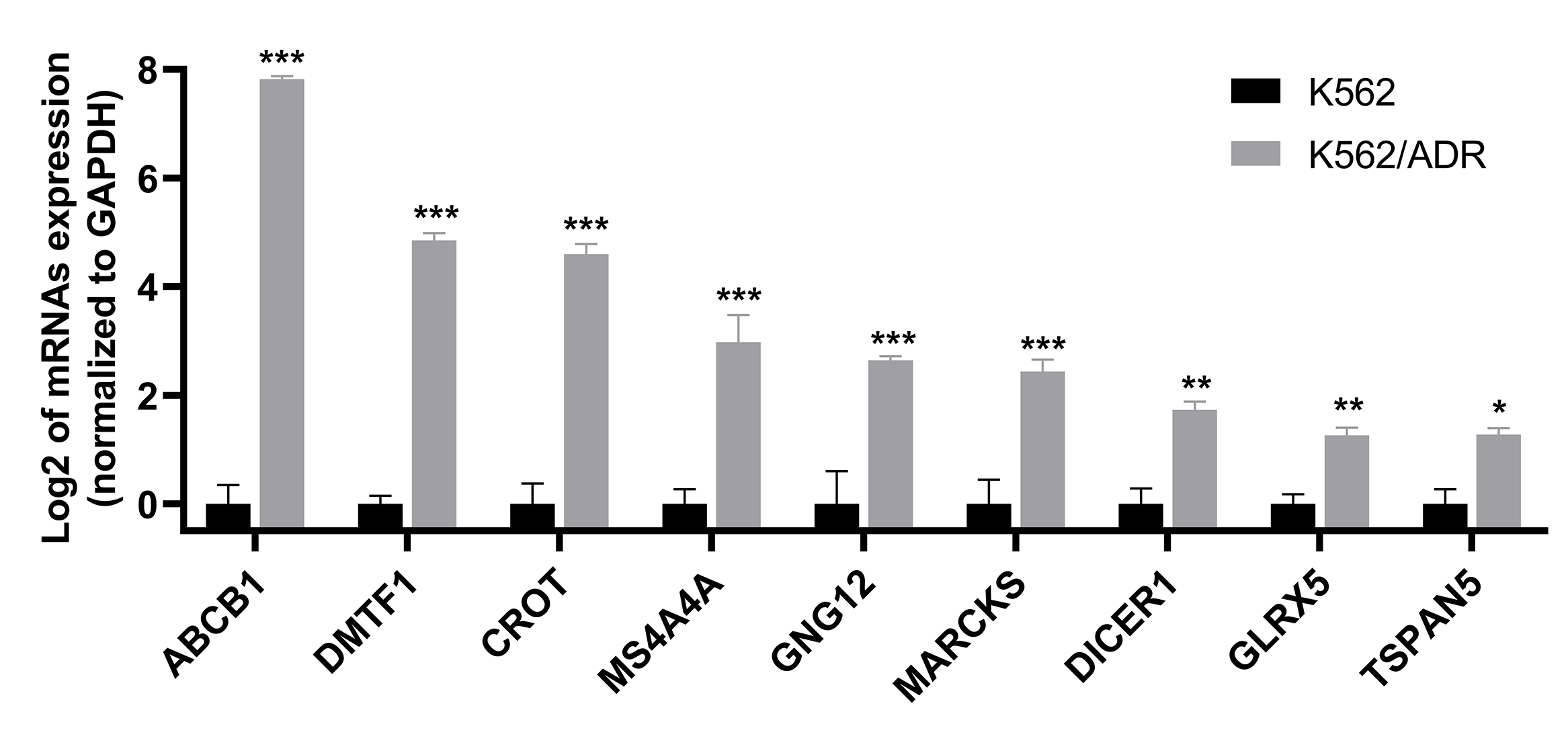

Supplement: Supplemental Information 4 — All data are represented as mean ± SD for at least three independent experiments. *P < 0.05, **P < 0.01 and ***P < 0.001 were statistically significant compared with the control group. [file peerj-09-11429-s004.png]

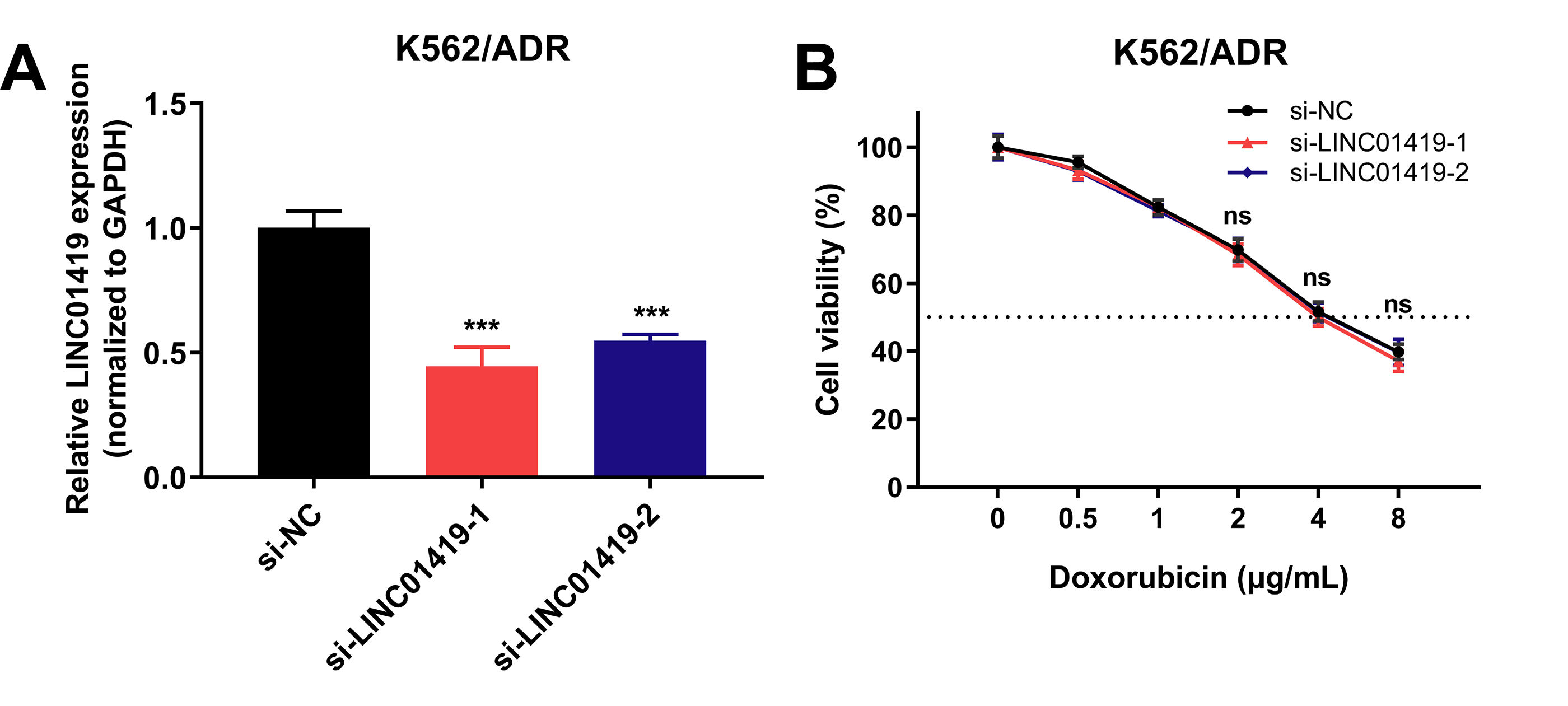

Supplement: Supplemental Information 5 — 48 hours after transfection with siRNAs in K562/ADR cells, LINC01419 expression was downregulated (A). Knockdown of LINC01419 did not affect the sensitivity of K562/ADR cells to doxorubicin (B). *P < 0.05, **P < 0.01 and ***P < 0.001 were statistically significant compared with the si-NC group. [file peerj-09-11429-s005.png]
